# Homeoprotein Msx1-PIASy Interaction Inhibits Angiogenesis

**DOI:** 10.3390/cells9081854

**Published:** 2020-08-07

**Authors:** Myung Jin Son, Seung Bae Rho, Kwangbae Kim, Mijung Oh, Chaeyeon Son, Sang Yong Song, Kyoungsook Park

**Affiliations:** 1 Stem Cell Convergence Research Center, Korea Research Institute of Bioscience and Biotechnology, Daejeon 34141, Korea; mjson@kribb.re.kr; 2 Division of Translational Science, Research Institute, National Cancer Center, Goyang 10408, Korea; sbrho@ncc.re.kr; 3 Medical Research Center, Sungkyunkwan University School of Medicine, Suwon 16419, Korea; ps2148@naver.com (K.K.); mijung.oh1@gmail.com (M.O.); snicki0203@gmail.com (C.S.); 4 Department of Pathology and Translational Genomics, Samsung Medical Center, Sungkyunkwan University School of Medicine, Seoul 06351, Korea

**Keywords:** Msx1, PIASy, VEGF, angiogenesis, protein stabilization

## Abstract

Previously, we demonstrated that the homeoprotein Msx1 interaction with p53 inhibited tumor growth by inducing apoptosis. However, Msx1 can exert its tumor suppressive effect through the inhibition of angiogenesis since growth of the tumor relies on sufficient blood supply from the existing vessels to provide oxygen and nutrients for tumor growth. We hypothesized that the inhibition of tumor growth by Msx1 might be due to the inhibition of angiogenesis. Here, we explored the role of Msx1 in angiogenesis. Overexpression of Msx1 in HUVECs inhibited angiogenesis, and silencing of Msx1 by siRNA abrogated its anti-angiogenic effects. Furthermore, forced expression of Msx1 in mouse muscle tissue inhibited vessel sprouting, and application of an Ad-Msx1-transfected conditioned medium onto the chicken chorioallantoic membrane (CAM) led to a significant inhibition of new vessel formation. To explore the underlying mechanism of Msx1-mediated angiogenesis, yeast two-hybrid screening was performed, and we identified PIASy (protein inhibitor of activated STAT Y) as a novel Msx1-interacting protein. We mapped the homeodomain of Msx1 and the C-terminal domain of PIASy as respective interacting domains. Consistent with its anti-angiogenic function, overexpression of Msx1 suppressed the reporter activity of VEGF. Interestingly, PIASy stabilized Msx1 protein, whereas deletion of the Msx1-interacting domain in PIASy abrogated the inhibition of tube formation and the stabilization of Msx1 protein. Our findings suggest the functional importance of PIASy-Msx1 interaction in Msx1-mediated angiogenesis inhibition.

## 1. Introduction

Ovarian cancer is a leading cause of cancer-related death in women [1]. Peritoneal dissemination of ovarian cancer cells remains a major concern for cancer-related mortality in ovarian cancer patients, and intraperitoneal dissemination of ovarian cancer results from tumor angiogenesis and invasion [2,3]. Angiogenesis, the formation of new blood vessels from a preexisting vasculature, involves coordinated endothelial cell proliferation, migration, and tube formation. This process is a prerequisite for normal embryo development as well as tumor growth and a critical factor in metastatic spread of malignant cells [3,4,5,6,7]. The vascular endothelial growth factor (VEGF) and its mitogenic receptor VEGFR-2 (Flk-1), which is localized on endothelial cells, are important mediators of tumor angiogenesis, and VEGF is responsible for peritoneal vessel permeability leading to ascites development [8]. Ovarian tumor cells overexpressed VEGF_165_ and VEGF_121_, and VEGF expression provides a survival advantage to transformed epithelial cells in the ovary [9]. In addition, the matrix metalloproteinase (MMP) activity contributes to the increase in tumor metastatic and angiogenic potential by degrading the basement membrane to invade adjacent tissues [10]. Although accumulating clinical findings reveal that VEGF-targeted anti-angiogenic therapies did not benefit all cancer patients, suppression of angiogenesis remains a major focus in the treatment of ovarian cancer and has been suggested as a promising ovarian cancer therapeutic [11].

Homeoproteins orchestrate gene expression and regulate cell growth, proliferation, differentiation, cell-to-cell communication, and the apoptotic pathway during pattern formation in embryogenesis [12]. The Msx1 homeobox gene encodes a homeoprotein that functions as a transcriptional repressor through interactions with components of the core transcription complex as well as other homeoproteins (Dlx2, Pax9, Lhx6, Lhx8, Snail, Lef-1, Sp3, Prx1, and Pitx2). Furthermore, Msx1 interacts with H1b, binds to the core enhancer element of MyoD, and inhibits muscle differentiation through chromatin remodeling [13]. Interestingly, the physiological significance of Msx1 in angiogenesis was reported in a mouse model system. Msx1 is expressed in retina endothelial cells at artery branching sites, and in subpopulations of vascular smooth muscle cells and pericytes after birth in mice [14,15]. Furthermore, Msx1 expression in endothelial cells was required for arteriogenesis remodeling [16]. Msx1 expression in highly vascularized tissues after birth in both human and mouse normal tissues as well as the requirement of Msx1 expression during an arteriogenic remodeling response suggested its involvement in angiogenesis. Our previous findings demonstrated that Msx1 exerts its function through interactions with the p53 tumor suppressor. We have shown that Msx1 inhibits tumor growth by inducing apoptosis in cervical cancer [17]. Tumor growth over a certain size requires a sufficient supply of oxygen and nutrient supply by existing vessels for the tumor to overcome the diffusion limitation of oxygen and nutrient [6]. However, the function of Msx1 in tumor angiogenesis has not been reported. Here, we explored the role of Msx1 in angiogenesis and characterized its anti-angiogenic function using gene overexpression and gene silencing approaches in vitro and in vivo. We also identified a protein inhibitor of activated STAT Y (PIASy) as an Msx1-interacting protein and demonstrated its functional importance in Msx1 protein stabilization via protein-protein interaction in the suppression of angiogenesis. Taken together, our results demonstrate a previously unknown function for Msx1 as an angiogenesis inhibitor in vitro and in vivo.

## 2. Materials and Methods

### 2.1. Cell Culture

HUVECs (Human umbilical vein endothelial cells) (Lonza, Basel, Swiss) were grown on 0.3% gelatin (Sigma-Aldrich, St. Louis, MO, USA)-coated dishes using the EGM-2 BulletKit (Lonza). Human ovarian cancer 2774 cells were obtained from the American Type Culture Collection (ATCC, Manassas, VA, USA) and cultured in DMEM containing 10% FBS and antibiotics (Life Technologies, Carlsbad, CA, USA).

### 2.2. Recombinant Adenoviral Constructs and Infection

The adenovirus vector expressing Msx1 (Ad-Msx1) was constructed according to a protocol described previously [3,17]. Briefly, a high-fidelity polymerase chain reaction (PCR)-amplified full-length mouse Msx1 cDNA fragment was cloned into *Hind*III/*Xho*I sites of the adenoviral plasmid shuttle vector p∆ACMVp(A) [3]. The sequence of the cloned mouse Msx1 cDNA was confirmed by DNA sequence analysis [3]. The resulting adenoviral vector was transfected into human embryonic kidney 293 cells for adenovirus production, as previously described [3,17]. Large batches of recombinant adenovirus were purified by centrifugation through two consecutive cesium chloride gradients [3]. Adenovirus containing an empty shuttle vector was used as a control [3].

### 2.3. Expression Constructs

The human Msx1-expression construct Flag-Msx1 was prepared by inserting full-length Msx1 cDNA obtained from Origene into the *Eco*RI-*Xba*I cloning site of the 3x-Flag vector (Clontech, Palo Alto, CA, USA) in a frame and then it was amplified and purified through a Qiagen plasmid large purification column. The mouse Msx1-expression construct pCB6-Msx1 was kindly provided by Professor Abate Shen (Robert Wood Johnson Medical School, NJ, USA). Full-length and C-terminal deletion mutant PIASy (PIASyΔC) constructs were provided by Dr. Tae H. Chun [18]. The sequence of all plasmids in the mammalian vector was confirmed by DNA sequencing. 

### 2.4. Small Interfering RNA (siRNA) Transfection

The siRNA oligonucleotide sequence targeting Msx1 corresponding to nucleotides 199–217 in the human Msx1 sequence was synthesized in Dharmacon as was the scrambled siRNA as a negative control siRNA to eliminate the off-target effect of siRNA in the experiments [3]. HUVECs were then infected with Ad-Msx1 alone or Ad-Msx1 with Msx1 siRNA (100 nM) [3]. Total RNA was extracted using a Trizol solution according to the protocol provided by the manufacturer, and RT-PCR was performed to examine suppression of Msx1 mRNA expression.

### 2.5. [ ^3^H] Methylthymidine Incorporation Assay

To measure cell proliferation, uninfected or Ad-mock, Ad-Msx1, or Ad-Msx1+siRNA-infected HUVECs were stimulated with or without VEGF (10 ng/mL) for 24 h. One μCi/mL of [^3^H] methylthymidine (Amersharm, Little Chalfont, UK) was added to each well. The incorporated [^3^H] methylthymidine was measured by a liquid scintillation counter (Beckman Coulter, Brea, CA, USA).

### 2.6. Migration and Invasion Assays

Uninfected or Ad-mock, Ad-Msx1, or Ad-Msx1+siRNA-infected HUVECs were seeded onto Transwells (Costar, Washington, DC, USA) for the migration assay and onto Matrigel (BD Biosciences, San Jose, CA, USA)-coated Transwells for the invasion assay [3]. HUVECs were either treated or untreated with VEGF (25 ng/mL) for 24 h for the migration assay and for 30 h for the invasion assay, as previously reported [3,19]. HUVEC cells cultured in a 24-well plate for the indicated time period were fixed with ethanol, and then non-migrated or invading cells on the upper surface of the filter membrane were wiped off with a cotton swab. The remaining cells on the bottom surface of the membrane filter were fixed in 100% methanol and stained with hematoxylin and eosin, followed by three rinses in distilled water as described previously [3]. The number of migrated or invaded cells was counted under a light microscope, and mean values were determined [3]. Independent experiments were repeated three times, and the data shown are the mean ± standard deviation (SD) of triplicate samples.

### 2.7. Tube Formation Assay

Twenty-four wells were coated with Matrigel (10 mg/mL) and left for 30 min at 37 °C for polymerization. Uninfected or Ad-mock-, Ad-Msx1-, or Ad-Msx1+siRNA-infected HUVECs (1 × 10^5^ cells) were seeded onto the surface of the Matrigel. Cells were then incubated for 48 h with 10 ng/mL of VEGF. Tube formation was observed by phase-contrast microscopy and photographed at 40x magnification (BX51, Olympus). Tube lengths were quantified using the Inform (PerkinElmer, Waltham, MA, USA) analysis program. The data shown are the mean ± SD of triplicate samples, and independent experiments were repeated three times [20]. 

### 2.8. Ex Vivo Skeletal Muscle Angiogenesis Assay

An ex vivo angiogenesis assay was performed as described [21]. A novel ex vivo angiogenesis assay based on electroporation-mediated delivery of naked plasmid DNA to skeletal muscle was performed [21]. In brief, legs from 12-week-old BALB/c mice were shaved and depilated to expose the tibialis anterior muscle. The indicated DNA plasmid was injected into the tibialis anterior muscle with a 30-gauge insulin syringe. Thirty seconds after DNA injection, transcutaneous electric pulses were applied to the surface of the injection site using an ECM830 electroporator. The mice were sacrificed two days after electroporation to retrieve the muscle. The muscle was washed in PBS and placed in a 24-well plate containing 200 A1 of growth factor-reduced Matrigel (Becton-Dickinson, Bedford, MA, USA) and incubated at 37 °C for 30 min to solidify the gel. The plate was cultured at 37 °C under 5% CO_2_. An outgrowth of capillary-like structures was observed with an inverted microscope (Zeiss, Oberkoche, Germany) equipped with a digital photography system. The mean area of microvessels was measured with a 1300 × 1030 pixel image captured with a digital CCD camera (AxioCAM, Zeiss) and quantified using the ImageLab imaging software (MCM Design, Birkeroed, Denmark). 

### 2.9. In Vivo Angiogenesis Assay 

Fertilized white leghorn chicken eggs obtained from a local poultry farm were incubated in a MultiQuip Incubator (E2) at 37 °C with 60% humidity. A small window was made in the egg shell of each fertilized egg on day 3 of chick embryo development to detach the chorioallantoic membrane (CAM) layer from the egg shell, and concentrated cell lysates obtained from Ad-mock vector alone or AD-Msx1-infected 2774 ovarian cancer cells were applied to the CAM of chick embryos. Subsequently, the window was resealed with adhesive tape, and eggs were returned to the incubator until day 14 of development. The CAM was fixed, and the blood vessels were quantified by counting the number of blood vessel branch points under the microscope. Ten sections per chick embryo were used for quantitative analysis of angiogenesis per treatment. 

### 2.10. Yeast Two-Hybrid (Y2H) Analysis and Quantitation of Interaction

The pGilda/LexA-human Msx1 fusion protein was constructed and used to screen binding proteins from a human ovary cDNA library (Clontech). A total of 4.5 × 10^6^ transformants were screened for potential interacting proteins. The binding proteins were expressed as B42 fusion proteins. Positive interactions were confirmed by cell growth on a leucine-depleted yeast synthetic medium and blue colony formation on a 5-bromo-4-chloro-3-indolyl-β-D-galactoside (X-gal, 5 M)-containing medium. The binding activity of the interaction was calculated using an ONPG β-galactosidase analysis system as described in two previous studies [22,23]. Furthermore, three deletion fragments (Met^1^-Leu^160^, Arg^161^-Gln^225^, and Glu^226^-Thr^297^) of Msx1 were introduced into the pGilda/LexA vector at the *Bam*HI and *Xho*I enzyme sites, respectively. The human protein inhibitor of activated STAT Y (PIASy) was cloned with cDNA encoding a full-length gene into the multi-cloning sites (MCS) of the pJG4-5 yeast shuttle plasmid, which included B42 fusion proteins (Clontech). Three truncated mutants (Met^1^-Arg^180^, Glu^181^-Ser^414^, and Cys^415^-Cys^510^) were cloned to the pJG4-5 plasmid to establish B42 fusion proteins at *Eco*RI and *Xho*I sites. The primers used to clone all constructs are presented in Table 1.

### 2.11. Immunoblotting and Co-Immunoprecipitation

Whole cell lysates from the Ad-Msx1-infected 293 cells transiently transfected with Flag-PIASy were lysed in a radioimmunoprecipitation assay (RIPA) buffer (50 mM Tris-HCl (pH 8.0), 150 mM NaCl, 1% NP-40, 0.1% sodium dodecyl sulfate (SDS), and 10 mM sodium deoxycholate) as previously described [17] and then incubated with anti-Msx1 antibody overnight at 4 °C. Immunocomplexes were immunoprecipitated using protein A/G agarose beads (Santa Cruz Biotechnology, Dallas, TX, USA) for 2 h at 4 °C with gentle stirring. The beads were washed three times with a lysis buffer and boiled in 50 μL of a 1x SDS sample buffer for 5 min at 95 °C. After centrifugation, the precipitated proteins were separated by SDS-PAGE and electrophoretically transferred onto an enhanced chemiluminescence (ECL) nitrocellulose membrane (GE Healthcare, London, UK). Then, co-immunoprecipitated Msx1 was detected by Western blot with anti-Flag antibody and visualized by chemiluminescence (Amersham) as described previously [19]. To analyze Msx1 protein stabilization by PIASy, human ovarian 2774 cells were co-transfected with Msx1-expressing plasmid and the indicated amount of Flag-PIASy plasmid. Protein lysates were prepared in a RIPA buffer and then subjected to SDS-PAGE, followed by immunoblotting with anti-Msx1 antibody. Equal protein loading was confirmed by sequential incubation of the membrane with anti-β-actin antibody. 

### 2.12. Confocal Immunofluorescence Microscopy

Cells grown on coverslips in 6-well plates were transfected with plasmids expressing GFP-Msx1 and Flag-PIASy. At 30 h after transfection, cells were washed with 1x PBS, fixed in 4% paraformaldehyde in 1x PBS, and then processed for indirect immunofluorescence microscopy. The anti-Flag antibody was used to detect PIASy, followed by incubating with Alexa 568 (Molecular Probes, Eugene, OR, USA) goat anti-mouse IgG as a secondary antibody. DAPI was used for nuclear staining. Expression and localization of the PIASy and Msx1 proteins were analyzed using a confocal microscope and photographed at 20x magnification (LSM700, Zeiss).

### 2.13. Transient Transfection and Luciferase Assays

The human VEGF promoter luciferase reporter constructs were described previously [24]. HUVECS at 70% confluency were transiently co-transfected with Ad-Msx1, Flag-PIASy, or Flag-PIASyΔC as indicated along with the VEGF reporter constructs using an Effectene transfection reagent (Qiagen, Hilden, Germany) according to the manufacturer’s instructions. As an internal control to correct for variations in transfection efficiency, 20 ng of pRL-TK (Promega, Madison, WI, USA) was co-transfected. Luciferase activity was measured using a dual luciferase reporter assay system (Promega) according to the manufacturer’s instructions and was normalized to *Renilla* luciferase activity to correct for variations in transfection efficiency.

### 2.14. Statistical Analysis

The statistical significance of differences in the data was evaluated by Student’s t-tests, and *p* < 0.05 was considered statistically significant.

## 3. Results

### 3.1. Msx1 Inhibits Endothelial Cell Migration, Invasion, and Tube Formation In Vitro

**Figure 1:**
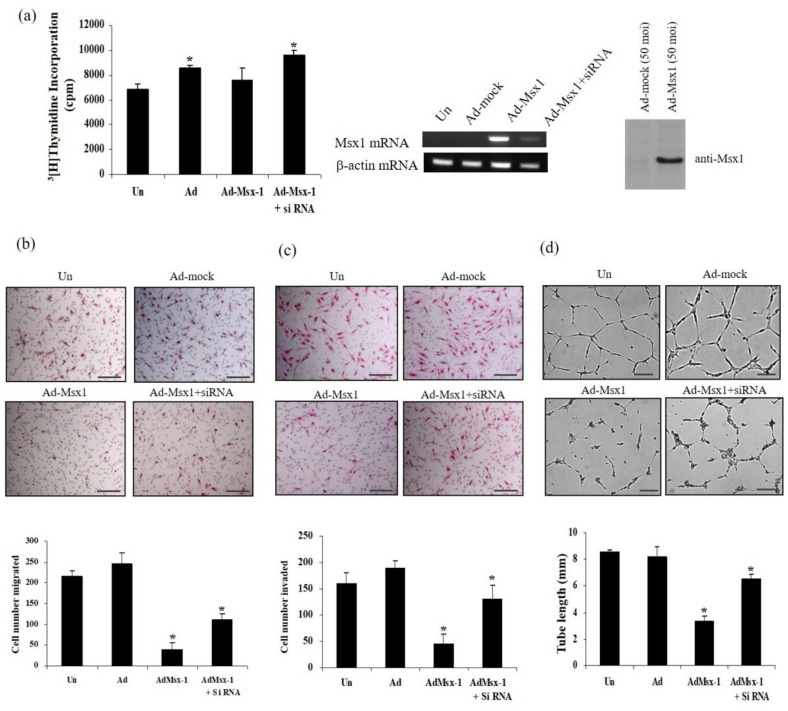
Msx1 inhibits endothelial cell migration, invasion, and tube formation in vitro. (**a**) siRNA-mediated suppression of Msx1 in HUVECs. HUVECs were either uninfected (Un) or infected with Ad-Mock, Ad-Msx1, or Ad-Msx1 with siRNA for Msx1 (Ad-Msx1+siRNA) for 18 h and then treated with the vascular endothelial growth factor (VEGF) (10 ng/mL) for 24 h. Expression of Msx1 was confirmed by RT-PCR and immunoblotted with anti-Msx1 antibody. Incorporated [^3^H]-thymidine was determined by liquid scintillation counting. (**b** and **c**) Uninfected HUVECs and indicated adenovirus-infected HUVECs were seeded onto Transwells for migration assay (**b**) or on Matrigel-coated Transwells for invasion assay (**c**), followed by stimulation with VEGF (25 ng/mL) for 24 (**b**) or 30 h (**c**), respectively. The number of migrated or invaded cells was counted under a light microscope, and mean values were determined. Independent experiments were repeated three times, and error bars correspond to 95% confidence intervals. * *p* < 0.05 compared to uninfected controls. (**d**) Uninfected HUVECs and adenovirus-infected HUVECs were plated on growth factor-reduced Matrigel and then treated with or without VEGF (10 ng/mL) for 48 h. Formation of tubular structures was detected by an inverted microscope. Tube lengths were quantified, and error bars correspond to 95% confidence intervals. The scale bar represents 100 μm * *p* < 0.05 indicates a significant difference.

To explore the role of homeobox protein Msx1 on tumor angiogenesis, which is essential for tumor growth, we examined the effects on VEGF-induced proliferation, migration, invasion, and tube formation in human endothelial cells (Figure 1). Overexpression of Msx1 did not inhibit VEGF-induced DNA synthesis in HUVECs and did not show a cytotoxic effect on normal endothelial cells (Figure 1a). In contrast, inhibition of Msx1 expression with Msx1-specific siRNA (Msx1-targeted small interfering RNA) slightly increased VEGF-induced DNA synthesis in HUVECs (Figure 1a). Next, we investigated whether overexpression of Msx1 regulated the effects of VEGF on endothelial cell migration and invasion. Transwell migration and invasion assays demonstrated that overexpression of Msx1 significantly reduced VEGF-induced migration and invasion of HUVECs (Figure 1b,c). Conversely, silencing of Msx1 by siRNA (Ad-Msx1+siRNA) failed to reduce VEGF-induced migration and invasion (Figure 1b,c). To confirm that Msx1 has direct anti-angiogenic effects, we examined endothelial tube formation by overexpression of Msx1. Uninfected or mock adenovirus (Ad-mock)-infected cells incubated with VEGF formed an organized network of endothelial cells on Matrigel (Figure 1d). In contrast, overexpression of Msx1 markedly inhibited VEGF-induced tube formation. Importantly, this inhibitory effect of Msx1 on VEGF-induced tube formation was abrogated by silencing of Msx1 expression due to the Msx1 siRNA treatment. These observations suggest the functional significance of Msx1 in angiogenesis in vitro.

### 3.2. Msx1 Inhibits Vessel Sprouting Ex Vivo and Angiogenesis In Vivo

**Figure 2:**
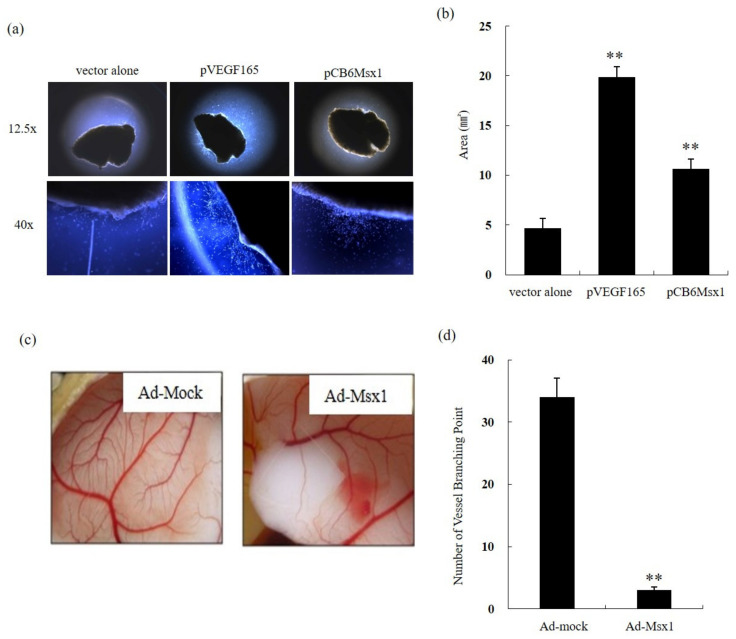
Msx1 inhibits vessel sprouting ex vivo and in vivo angiogenesis. (**a**) Cross-sections of a mouse tibialis anterior muscle injected with pVEGF165 or pCB6 Msx1 plasmid by electroporation were embedded in growth factor-reduced Matrigel. An outgrowth of capillary-like structures was observed with an inverted microscope. (**b**) The outgrowth was quantitated by measuring the area of capillary-like structures. (**c**) Ad-Msx1 inhibits angiogenesis in vivo. Ten-day-old chicken chorioallantoic membranes (CAMs) were treated with lysates obtained from cells infected with either Ad-β-gal or Ad-Msx1 virus. After 72 h, CAMs were harvested, and vascular density was assessed by microscopy. (**d**) Angiogenesis was quantitated by counting the number of branch points arising from the pre-existing vessels. Results were from three independent experiments. ** *p* < 0.01 indicates a significant difference.

To evaluate whether Msx1 inhibits vessel sprouting, an ex vivo explant assay was performed. Abundant vessel sprouting was detected in vector-transfected explants in the presence of VEGF (Figure 2a, middle). In contrast, overexpression of Msx1 showed dramatically reduced VEGF-induced vessel sprouting (Figure 2a, right). Quantitation analysis showed that overexpression of Msx1 inhibited vessel sprouting 2.8-fold (Figure 2b). To further confirm the above results in vivo, a CAM assay using 10-day-old CAMs was performed with the concentrated whole cell lysates obtained from Ad-mock- or Ad-Msx1-treated 2774 cells. Consistent with our ex vivo assays, Msx1 overexpression markedly inhibited vessel branching in vivo (Figure 2c). Quantitation analysis demonstrated that overexpression of Msx1 inhibited vessel branching by >10-fold (Figure 2d). These findings suggest that Msx1 suppresses capillary formation ex vivo and in vivo. 

### 3.3. Identification of PIASy as a Novel Msx1-Interaction Protein

**Figure 3:**
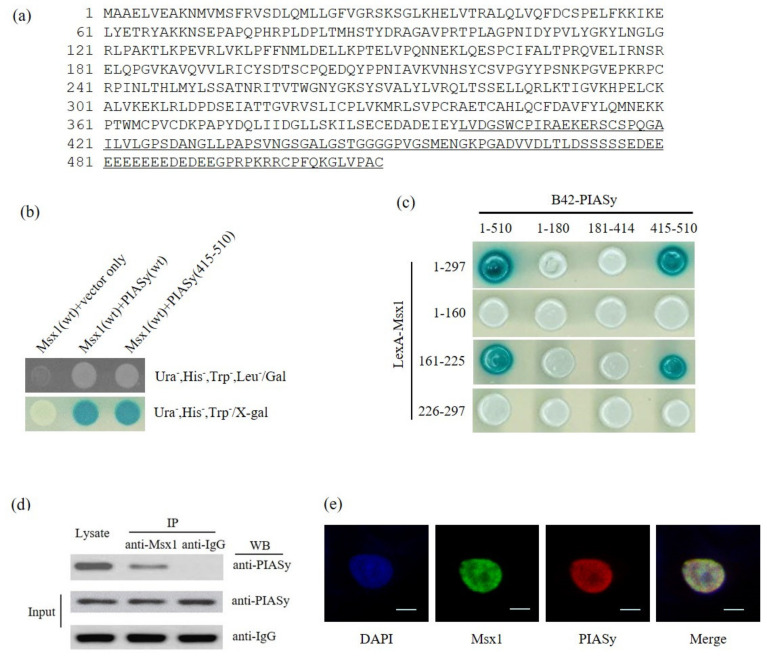
Homeoprotein Msx1 interacts with PIASy. (**a**) Identification of PIASy as Msx1-interacting protein by yeast 2-hybrid (Y2H) screening. The amino acid sequence of PIASy is presented using single letter abbreviations. The underlined amino acid sequence is the translated PIASy protein isolated from the yeast two-hybrid (Y2H) assay. Msx1 and PIASy cDNA constructs were introduced into EGY48 yeast host strains to test for protein-protein interactions within the Y2H analysis system. Transformants were examined for their ability to grow on a medium lacking leucine at 30 °C (**b**, upper panel) and for β-galactosidase expression (b, bottom panel). (**c**) Biological interaction between cDNA constructs for three truncated Msx1 (Met^1^-Leu^160^, Arg^161^-Gln^225^, and Glu^226^-Thr^297^) and three deleted PIASy (Met^1^-Arg^180^, Glu^181^-Ser^414^, and Cys^415^-Cys^510^) fusion proteins in the Y2H analysis system. (**d**) Interaction of Msx1 and PIASy in mammalian cells. HEK 293 cells were infected with Ad-mock or Ad-Msx1 and then transiently transfected with Flag-PIASy harboring a full-length PIASy. The whole cell lysates were used for immunoprecipitation with anti-IgG or anti-Msx1 antibody, followed by immunoblotting with anti-Flag antibody. Expression of Msx1 was confirmed by immunoblotting with anti-Msx1 antibody. (**e**) Nuclear co-localization of Msx1 and PIASy in human ovarian cancer cells. Human ovarian cancer 2774 cells were co-transfected with pEGFP-Msx1 expressing a full-length Msx1 and Flag-PIASy expressing a full-length PIASy and then subjected to indirect immunofluorescence staining with Alexa Fluor 568-conjugated secondary antibody. The subcellular co-localization of Msx1-PIASy was examined using confocal microscopy. The co-localization of EGFP-Msx1 and Flag-PIASy is shown in the merged images. Scale bar, 10 μm.

To understand the mechanism involved in the inhibition of angiogenesis by Msx1, we screened for Msx1-binding proteins using a yeast two-hybrid (Y2H) assay. Approximately 4.5 × 10^6^ independent transformants were pooled. After re-spreading on selection media (Ura^−^, His^−^, Trp^−^, and Leu^-^), we observed nine colonies; a total of four colonies showed galactose dependency. The plasmids were collected from the selected yeast cells and transformed into *E. coli* KC8 to separate those carrying pJG4-5/B42-cDNA inserts. The plasmid DNAs were then isolated according to the previously described protocols [12], and the purified plasmid cDNAs were sequenced. A homology search in GenBank using the BLAST program showed that all four plasmid cDNAs encoded a human protein inhibitor of activated STAT Y (accession number: NM_015897; Figure 3a). A positive interaction between Msx1 and PIASy was verified both by cell growth (Figure 3b, upper) and a β -galactosidase assay (Figure 3b, bottom). An empty-inserted plasmid (vector only) was used as the negative control. To further confirm and identify the interacting domains between two proteins, both PIASy and Msx1 deletion mutants expressing the indicated domain were generated and used in the Y2H β-galactosidase assay (Figure 3c). We identified the homeodomain of the Msx1 protein as a PIASy interaction domain. In contrast, a deletion mapping study revealed that the C-terminal region (415–510aa) of PIASy, which contained the ring finger domain and the Ser/Ac region, was the Msx1-interacting domain. Finally, the interaction between the Msx1 homeodomain (161–225aa) and the C-terminal region of PIASy (415–510aa) was confirmed with the Y2H assay. Consistent with this result, the C-terminal region of PIASy (415–510aa) was initially identified from the library screening (Figure 3a).

Next, to confirm this interaction in mammalian cells, Ad-Msx1 containing a full-length Msx1 was infected in the presence of the PIASy expression plasmid. Consistent with the interaction results in a yeast system, a co-immunoprecipitation assay showed an interaction between Msx1 and PIASy in mammalian cells (Figure 3d). Furthermore, we investigated their interaction by confocal imaging analysis after co-transfecting human 2774 ovarian cultured cells with GFP-Msx1 and Flag-PIASy containing a wild-type full-length transcript of the respective gene. Msx1 was co-localized with PIASy in the nucleus as expected for a transcription factor (Figure 3e), revealing their interaction in mammalian cells. 

### 3.4. Msx1-PIASy Interaction is Necessary for Msx1-Mediated Angiogenesis

**Figure 4:**
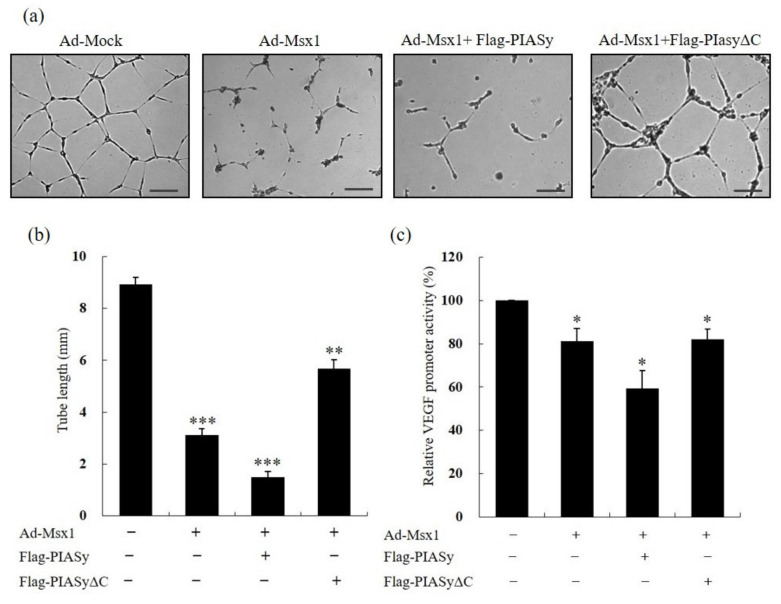
Homeoprotein Msx1-PIASy interaction is critical for suppression of angiogenesis in vitro. (**a**) PIASy-Msx1 interaction is crucial for angiogenesis in vitro. Adenovirus-infected HUVECs were plated on growth factor-reduced Matrigel and then transfected with the indicated PIASy construct expressing full-length (PIASy) or C-terminal-deleted PIASy (PIASyΔC) that did not contain the Msx1-interacting domain in the presence of VEGF (10 ng/mL) for 48 h, and tube formation was assessed. The formation of tubular structures was detected by an inverted microscope. Tube length was quantified by measuring the length of tubular structures. The results are expressed as the mean value ± standard deviation (SD). The scale bar represents 100 μm. (**b**) Quantification of the tube length. Tube lengths were quantified, and error bars correspond to 95% confidence intervals. ** p < 0.01, *** p < 0.001 indicates a significant difference. (**c**) Msx1-PIASy interaction is critical for transcriptional repression of the VEGF promoter in HUVECs. HUVECs were co-transfected with the indicated PIASy construct and the luciferase reporter construct containing the human VEGF promoter sequence. After 18 h, co-transfected HUVECs were further infected with Ad-mock or Ad-Msx1 for an additional 24 h. Total cell lysates were prepared and subjected to a dual luciferase assay to determine VEGF promoter activity, and relative luciferase activity was plotted. * p < 0.05 indicates a significant difference.

In an attempt to understand the significance of a PIASy-Msx1 interaction for angiogenesis, we next determined whether the PIASy-Msx1 interaction is important for the angiogenesis inhibitory function of Msx1. HUVECs were co-transfected with both Ad-Msx1 and an indicated PIASy expression vector, and formation of tubular structures was examined. Msx1 inhibited tube formation, and the addition of wild-type PIASy further suppressed tube formation (Figure 4a). Quantitation analysis demonstrated that overexpression of full-length Msx1 and PIASy inhibited tube formation 6-fold (Figure 4b). In contrast, overexpression of the deletion mutant PIASy (PIASyΔC), which did not contain the C-terminal region (415–510aa) of PIASy that we identified as the Msx1 interaction domain, failed to inhibit tube formation. Since Msx1 is a transcriptional factor, we examined the effect of the PIASy-Msx1 interaction on the transcriptional activity of the major angiogenesis regulator, VEGF, in HUVECs. Forced expression of Msx1 in HUVECs repressed VEGF transcriptional activity by 20%. The presence of PIASy further repressed VEGF transcriptional activity by 40% (Figure 4c). In contrast, transfection with an Msx1-interaction-defective PIASy mutant (PIASyΔC) failed to further repress the VEGF promoter activity. Taken together, our data suggest that the Msx1-PIASy interaction plays a critical role in the angiogenesis inhibitory function of Msx1 in vitro.

### 3.5. Msx1-PIASy Interaction Is Critical for Msx1 Protein Stabilization 

**Figure 5:**
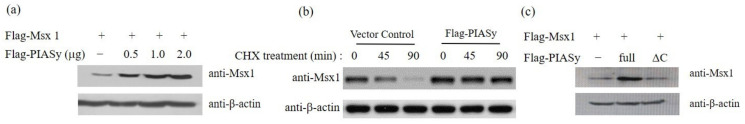
Msx1 protein stabilization by PIASy. (**a**) PIASy stabilizes Msx1 protein. Human ovarian cancer 2774 cells were co-transiently transfected with plasmid as indicated, and whole cell lysates were prepared and processed for immunoblot analysis with the indicated antibody. β-actin was used as a control. (**b**) PIASy increases the half-life of Msx1 protein. Human ovarian cancer 2774 cells were transiently transfected with the vector control or Flag-PIASy plasmid as indicated, and cycloheximide was added at the indicated time point. Whole cell lysates were prepared for immunoblot analysis with anti-Msx1 antibody. β-actin was used as a control. (**c**) C-terminal PIASy domain is critical for PIASy-mediated Msx1 protein stabilization. Human ovarian cancer 2774 cells were transfected with wild-type PIASy (full) or mutant PIASy construct lacking Msx1-interacting C-terminal domain (ΔC) and then processed for immunoblot analysis with the indicated antibody to assess the stabilization of Msx1 protein. β-actin was used as a control.

To further examine the functional significance of PIASy as an Msx1-interacting protein and its impact on the anti-angiogenic function of Msx1, we investigated whether PIASy is involved in the stabilization of Msx1. Forced expression of PIASy stabilized the Msx1 protein in a concentration-dependent manner (Figure 5a). Next, since the intracellular concentration of Msx1 can primarily be regulated by modulation of its stability, we compared the kinetics of Msx1 degradation after CHX treatment in cells transfected with a control vector or full-length PIASy expression vector. Densitometric scanning showed that the half-life of Msx1 protein in control vector-transfected cells was < 60 min, whereas PIASy-overexpression led to a significant increase in the half-life of Msx1 protein (Figure 5b). This strongly suggests that PIASy overexpression can stabilize Msx1 protein and subsequently enhance Msx1-mediated angiogenesis inhibition. To further confirm the significance of PIASy-Msx1 interaction for stabilization of Msx1 protein, a deletion mutant PIASy (PIASyΔC) without the C-terminal region (415–510aa) of PIASy identified in the Msx1 interaction was tested for its ability to stabilize Msx1. The PIASy deletion mutant (PIASyΔC) failed to stabilize the Msx1 protein (Figure 5b). This result is consistent with the critical function of PIASy-Msx1 interaction in inhibiting angiogenesis as demonstrated above (Figure 4). These observations confirmed that the Msx1-PIASy interaction is critical for Msx1 stabilization, and that PIASy-mediated Msx1 stabilization is functionally significant in Msx1-mediated angiogenesis inhibition. 

## 4. Discussion

In our previous experiments, we found a dramatic reduction in tumor size by Msx1 overexpression [17]. The growth of tumor requires enhanced cell proliferation as well as a sufficient blood supply; otherwise, tumor growth cannot be maintained due to the diffusion limitation of oxygen and nutrients. Thus, we thought that the possibility of a novel function of Msx1 in the inhibition of tumor growth would involve regulation of angiogenesis and initiated the function of Msx1 in angiogenesis. We demonstrated a novel angiogenesis inhibitory function of homeoprotein Msx1 using gene overexpression and gene silencing approaches in vitro and in vivo. Importantly, overexpression of Msx1 at the dose used here did not inhibit VEGF-induced DNA synthesis of HUVECs and did not show cytotoxic effects on normal endothelial cells (Figure 1a). This normal cell sparing effect is critical for selecting an anti-angiogenic factor as a potential anti-cancer therapeutic. Furthermore, ex vivo and in vivo assays confirmed the marked angiogenesis inhibitory effect of Msx1 (Figure 2). Our previous report characterized Msx1 as a potential repressor of cell cycle progression, as evidenced by a marked increase in the length of the G1 phase of the cell cycle in cancer cells [25]. Consistent with this result, we observed a dramatic suppression of cyclin D1, D3, and E, along with a suppression of cyclin-dependent kinase 4 (CDK 4), c-Jun, and Rb proliferation associated protein gene. In contrast, we found an elevated expression of the genes involved in growth arrest and apoptosis (GADD153 and apoptotic cysteine protease MCH4) detected by cDNA expression array analysis [25]. Previously, we have demonstrated a novel function of Msx1 as a regulator of the p53 tumor suppressor in human tumors. Our results showed that Msx1 can exert its tumor suppressive effect by inducing apoptosis. However, Msx1 can exert its tumor suppressive effect by inhibiting angiogenesis [17]. Since VEGF production is elevated in many tumors including ovarian cancer, therapeutic strategies targeting VEGF and VEGF receptor signaling can inhibit angiogenesis and have been tested in clinical trials [26]. However, clinical results have not been satisfactory due to the fatal side effects of targeted angiogenesis inhibitors [27,28]. To identify a novel angiogenesis regulator, we recently reported a non-proteolytic caspase, calpain-6, as a novel VEGF-interacting partner and demonstrated its role in angiogenesis [19].

We also saw that forced overexpression of Msx1 in 2774 ovarian cancer cells with downregulated Msx1 expression reduced the expression of endogenous VEGF mRNA and its protein compared with control vector expression cells (data not shown). Consistent with the downregulation of VEGF mRNA by Msx1 overexpression, forced expression of Msx1 significantly repressed VEGF reporter activity (Figure 4c) and VEGF protein (see Supplementary Figure S3).

Several homeobox genes have been reported to regulate metastasis and angiogenesis. For example, overexpression of homeobox gene HoxD3 induced an angiogenic phenotype [29] and induced coordinate expression of metastasis-related genes in human lung cancer cells [30]. Homeobox B3 also promoted capillary morphogenesis and angiogenesis [31]. In contrast, sustained expression of homeobox D10 inhibited angiogenesis [32]. Furthermore, HoxB7 is reported to be a key factor for the tumor-associated angiogenic switch [33]. Based on our results, homeobox Msx1 can be added to the list of potential angiogenesis inhibitory homeobox genes.

We identified PIASy as a novel Msx1-interacting protein by yeast 2-hybrid (Y2H) screening and confirmed their interaction in mammalian cultured cells by immunoprecipitation and confocal imaging analyses (Figure 3). Co-localization of Msx1 and PIASy in the nucleus suggests a pivotal role in transcriptional repression of Msx1 target genes. As expected, we noted downregulation of VEGF mRNA and VEGF reporter activity by forced expression of Msx1 (Figure 4c and data not shown).

PIASy is a member of the PIAS family that acts as nuclear matrix-associated SUMO E3 ligases and is known to repress transcriptional activity [34,35,36]. PIASy was reported to be highly expressed in endothelial cells, enhanced SUMO conjugation to GATA-2, and suppressed endothelin-1 (ET-1) promoter activity in endothelial cells [18]. Overexpression of PIASy led to an increase in the stability of endogenous and ectopically expressed Ets-1 protein by preventing proteasomal degradation. 

The RING domain of PIASy has been identified as an important functional determinant of ubiquitination and SUMO E3 ligases. PIASy controls ubiquitination-dependent proteasomal degradation of Ets-1 [37,38]. PIASy could also inhibit angiogenesis through degradation of hypoxia-inducible factor-1α by SUMOylation, which is an independent function of Msx1 stabilization [39]. Furthermore, the RING domain of PIASy was reported to be involved in direct physical interaction with Smads and mediates sequestration of Smad1 in nuclear bodies. PIASy-Smads interaction suppressed TGF-β signaling [40]. In contrast, in mouse C2C12 myoblast cells, the interaction of Msx1 with PIAS1 was required for transcriptional repression and inhibition of myoblast differentiation [41]. Other reported functions of Msx1 homeoprotein include repression of the glycoprotein hormone α subunit gene by interaction of the Msx1 homeodomain and TATA-binding protein [42]. In contrast, interaction of the C-terminal domain of Msx1 with a heat shock factor led to activation of *Hspa1b* promoter activity [43].

To identify the VEGF receptor involved in Msx1-PIASy-mediated angiogenesis suppression, we performed a yeast-2-hybrid assay to explore which VEGF receptor interacted with Msx1. We found that only VEGFR2 interacted with Msx1 in yeast. To further confirm their interaction in mammalian cells, 293T cells were transfected with Flag-Msx1 and pcDNA-VEGFR1 or pcDNA-VEGFR2 expression vectors, and whole cell lysates were used for immunoprecipitation with anti-Flag (for Msx1), followed by immunoblot analysis with anti-VEGFR1 and anti-VEGFR2 antibody, respectively. We observed only the Msx1-VEGFR2 interaction in our immunoprecipitation in mammalian cells (Figure 2). Finally we addressed whether Msx1 inhibited angiogenesis in vivo using an ovarian xenograft model [3]. Human 2774 ovarian cancer cells tagged with a green fluorescence protein (GFP) were injected intraperitoneally into nude mice (n = 20 for each group), and then either Ad-mock or Ad-Msx1 was injected intraperitoneally. Injection of Ad-Msx1 suppressed the tumor growth significantly by measuring the tumor weight. To explore whether impaired tumor growth was associated with a paucity of neovasculature, frozen sections of the tumors were stained with CD31 antibody to detect endothelial cells. Tumors from Ad-mock-injected mice had abundant vasculature, whereas those from Ad-Msx1-treated mice had sparser vessels compared to their Ad-mock counterparts. These results were quantified by counting the vessel density. A statistically significant decrease in vessel density was achieved with an Ad-Msx1 treatment. We also compared expression of VEGF protein in serial frozen sections of the respective tumors by immunohistochemistry. Tumors derived from Ad-Msx1-treated mice showed a dramatic reduction in VEGF protein expression compared to those from Ad-mock mice. We observed a significant inhibition of tumor growth from the in vivo xenograft model (see Supplementary Figure S3).

**Figure 6:**
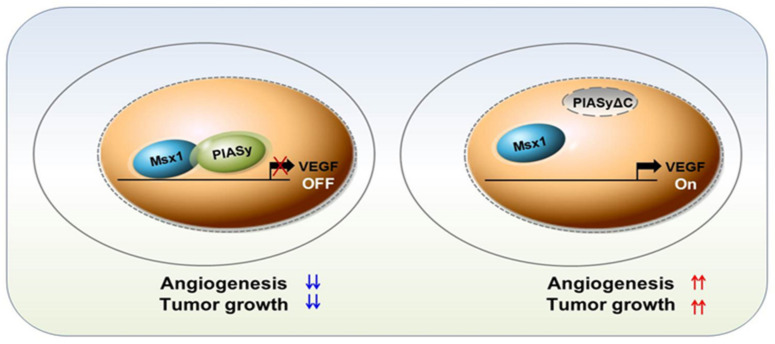
Diagram of proposed Msx1-PIASy-mediated angiogenesis suppression.

In summary, our findings demonstrate for the first time that homeoprotein Msx1 is a novel negative regulator of tumor angiogenesis (Figure 6). The PIASy-Msx1 interaction is critical for Msx1-mediated anti-angiogenic function by promoting VEGF transcriptional repression. Our findings revealed that Msx1 inhibits not only vessel formation in HUVECs via PIASy-mediated stabilization of Msx1, but also Msx1 represses a key angiogenic factor, VEGF, via transcriptional repression. A novel Msx1-interacting protein, PIASy, identified in this study can stabilize the homeoprotein Msx1 and promote the anti-angiogenic function of Msx1 by marked transcriptional suppression of human VEGF. Our results suggest that the homeoprotein Msx1 can be a potential therapeutic target for angiogenesis inhibition in pathological conditions including cancers. Taken together, these findings indicate that identification of Msx1 target genes and interacting proteins will lead to exploration of a novel function of Msx1 and will contribute to our understanding of the mechanism of Msx1-induced angiogenesis in physiological and pathological conditions.

## 5. Patents

Parts of this study have been patented in the United States (US20090264357A1 and US 9439944B2).

## Figures and Tables

**Table 1:** Primer sequences used for yeast two-hybrid (Y2H) analysis.

Gene	Forward primer (5’-3’)	Reverse Primer (5’-3’)
Msx1 (1-297)	cggggattcgtatgacttctttgccactc	attctcgagctatgtcaggtggtacat
Msx1 (1-160)	cggggattcgtatgacttctttgccactc	attctcgagttagagggtgcaggctgg
Msx1 (161-225)	attggattcgtcgcaaacacaagacgaac	cggctcgagttattgtagtctctttgc
Msx1 (226-297)	attggattcgtgaggcagagctggagaag	attctcgagctatgtcaggtggtacat
PIASy (1-510)	attgaattcatggcggcggagctggtg	attctcgagtcagcaggccggcaccag
PIASy (1-180)	attgaattcatggcggcggagctggtg	attctcgagtcacctggagttccggat
PIASy (181-414)	attgaattcgaactgcagcccggagtt	attctcgagtcagctgcgctccttttc
PIASy (415-510)	attgaattccgcagcccgcagggcgcc	attctcgagtcagcaggccggcaccag

## Supplementary Materials

The following are available online at https://www.mdpi.com/2073-4409/9/8/1854/s1, Figure S1: Identification of upstream and downstream signaling pathways involved in angiogenesis inhibition by Msx1 and PIASy, Figure S2: Msx1 protein can interact with VEGFR-2 but not with VEGFR-1, Figure S3: Msx1 suppressed in vivo tumor angiogenesis.
