# Supplementary material for: Homeoprotein Msx1-PIASy Interaction Inhibits Angiogenesis
Source: Cells. 2020 Aug 7;9(8):1854. doi: 10.3390/cells9081854 (PMC7463958; doi:10.3390/cells9081854)
Supplement: Supplementary file 1 [file cells-09-01854-s001.pdf]

Son *et al.* **Supplementary Materials**

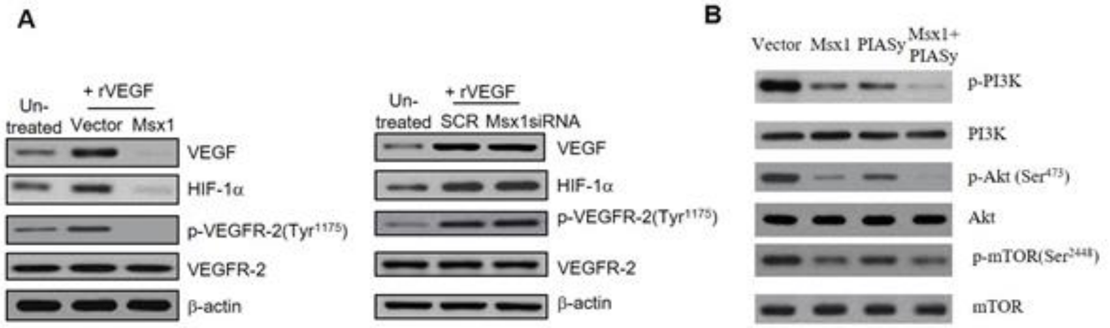

Figure S1. Identification of upstream and downstream signaling pathways involved in angiogenesis inhibition by Msx1 and PIASy. (A) Msx1 inhibits VEGF, HIF-1α, and VEGFR-2 phosphorylation. HUVEC cells were transfected with vector control or Msx1 expression plasmid and incubated with 10 ng/ml of recombinant VEGF. Total cell lysates were prepared and immunoblotted with the indicated antibody. Msx1 overexpression decreased VEGF protein, HIF-1α, and phosphorylated VEGFR2 (Tyr^{1175}) levels compared to the control vector-transfected HUVECs. Protein expression of both VEGF and HIF-1α was detected by immunoblotting. Total VEGFR-2 and β-actin were used as loading controls. To confirm the effect of Msx1 in VEGF signaling, HUVEC cells were transfected with scrambled siRNA (SCR) or Msx1siRNA using Lipofectamine RNAiMAX transfection reagent and then incubated with VEGF (10 ng/ml). Total cell lysates were prepared and immunoblotted with the indicated antibody. Overexpression of Msx1 suppressed the VEGF, HIF-1α, and p-VEGF receptor 2 (VEGFR-2). In contrast, silencing of Msx1 by Msx1siRNA failed to suppress VEGF, HIF-1α, and p-VEGFR-2 (Tyr^{1175}). Total VEGFR-2 and b-actin were included as loading controls. (B) Msx1 and PIASy led to inhibition of the downstream PI3K-mTOR-mediated signaling pathway. HUVEC cells were transfected with the indicated expression plasmids, and whole cell lysates were prepared for immunoblot analysis with the indicated antibody to examine the effect of MSx1 and PIASy in the downstream PI3K-mTOR-mediated signaling pathway. We observed marked reduction of phosphorylation of PI3K, Akt, and mTOR proteins. Antibodies to the non-phosphorylated PI3K, AKT, and mTOR proteins were used to verify equal protein loading.

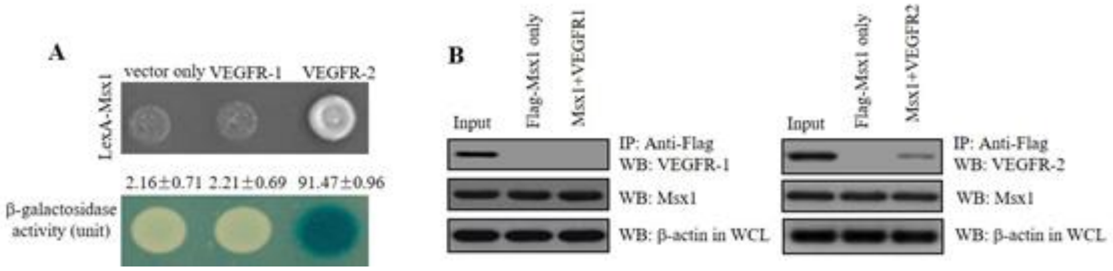

Figure S2. Msx1 protein can interact with VEGFR-2 but not with VEGFR-1. (A) Positive Msx1-VEGFR-2 interaction was examined by Y2H assay by growing yeast cells in medium lacking leucine and identified by the formation of the subsequent blue colonies on X-gal plates containing 2% galactose. Binding activity (unit) was calculated by adding *o*-nitrophenyl β-D-galactopyraniside (ONPG) and was shown below the corresponding lane. (B) Msx1 interacted with VEGFR-2 in human cells. Whole cell lysates from HEK 293T cells co-transfected with Flag-Msx1 plasmid or both VEGFR-1 and VEGFR-2 plasmids were immunoprecipitated with anti-Flag antibody and immunoblotted with either anti-VEGFR-1 or anti-VEGFR-2 antibody. β-actin was used as a loading control.

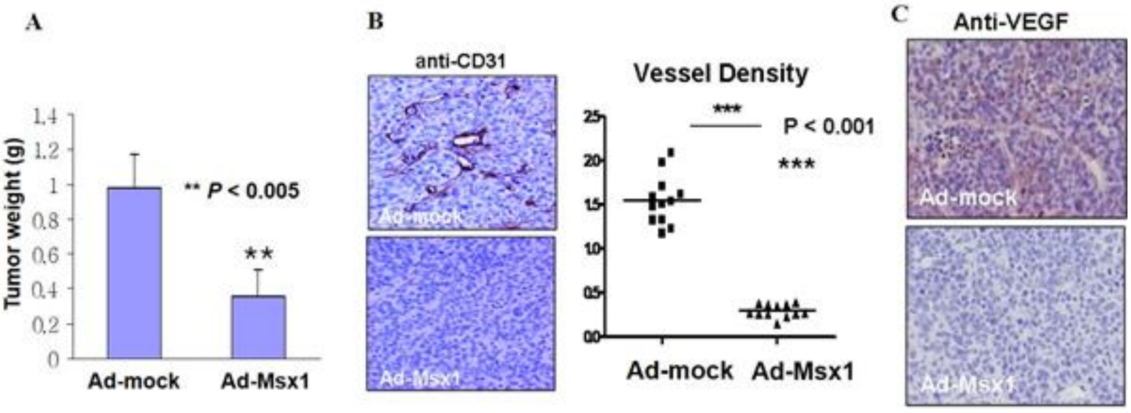

Figure S3. Msx1 suppressed *in vivo* tumor angiogenesis. (A) Msx1 suppressed tumor growth. Human 2774 ovarian cancer cells were injected intraperitoneally into nude mice, and then either Ad-mock or Ad-Msx1 was injected intraperitoneally. Injection of Ad-Msx1 suppressed tumor growth significantly as shown by measuring tumor weight. (B) To explore whether impaired tumor growth was associated with paucity of neovasculature, frozen sections of the tumors were stained with anti-CD31 antibody to detect endothelial cells by immunohistochemistry. These results were quantified by counting vessel density. A statistically significant decrease in vessel density was observed with Ad-Msx1 treatment. (**C**) To compare the expression of VEGF protein, serial frozen sections of the respective tumors were analyzed by immunohistochemistry with anti-VEGF protein. Tumors derived from Ad-Msx1-treated mice showed a

dramatic reduction in VEGF protein expression compared to those from Ad-mock mice. Tumor tissues derived from Ad-Msx1-treated mice showed dramatic reduction in tumor weight, vessel density, and VEGF protein expression compared to those from Ad-mock mice.
